# “Cultural brokerage” and beyond: piloting the role of an urban Aboriginal Mental Health Liaison Officer

**DOI:** 10.1186/s12889-015-2221-4

**Published:** 2015-09-10

**Authors:** Brian McKenna, Sabin Fernbacher, Trentham Furness, Michelle Hannon

**Affiliations:** School of Nursing, Midwifery and Paramedicine, Australian Catholic University, Fitzroy, Australia; NorthWestern Mental Health, Level 1 North, City Campus, The Royal Melbourne Hospital, Grattan Street, Parkville, VIC 3050 Australia; Northern Area Mental Health Service, Epping, Australia

## Abstract

**Background:**

Suboptimal use of mental health services persists for Aboriginal and Torres Strait Islander peoples^1^. Coupled with poorer life expectancy than other Australians, barriers to care have included poorly established partnership and communication among mental health services and Aboriginal peoples, and cultural insensitivity. As such, a goal of the Aboriginal mental health workforce is to engage their people and improve the social and emotional well-being of Aboriginal peoples. In 2013, the Northern Area Mental Health Service piloted a 0.8 full time equivalent position of an Aboriginal Mental Health Liaison Officer in an urban setting. Therefore, aims of this study were to describe the development of the role and stakeholder perceptions on how the role impacts on the typical journey of Aboriginal consumers engaging with mental health services. Meeting the aims may provide an exemplar for other mental health services.

**Methods:**

An illustrative case study using quantitative and qualitative data collection was undertaken. Descriptive statistics were computed to profile consumers and referral pathways. Thematic analysis was used to profile key stakeholder perceptions of the role.

**Results:**

The Aboriginal Mental Health Liaison Officer received 37 referrals over a 9 month period. The major source of referral was from an emergency department (49 %). Seventy-three percent of referrals by the Aboriginal mental health liaison officer at discharge were to community mental health teams. Thematic analysis of data on the development of the role resulted in two themes themes; (1) realisation of the need to improve accessibility and (2) advocating for change. The description of the role resulted in four themes; (1) the initiator: initiating access to the service, (2) the translator: brokering understanding among consumers and clinicians, (3) the networker: discharging to the community, and (4) the facilitator: providing cyclic continuity of care.

**Conclusions:**

The liaison component of the role was only a part of the multiple tasks the urban Aboriginal Mental Health Liaison Officer fulfils. As such, the role was positively described as influencing the lives of Aboriginal consumers and their families and improving engagement with health professionals in the mental health service in question.

## Background

A disparity exists in the health status between Aboriginal peoples and other Australians [1, 2]. Prior to the age of 65 years, the mortality rate of Aboriginal peoples is three times greater than other Australians [1]. Life expectancy of Aboriginal peoples is 71 years compared with 81 years for other Australians [3]. Furthermore, the hospitalisation rate for mental health problems of Aboriginal peoples aged between 15 and 54 years was 31 per 1000 population, compared with 18 per 1000 for other Australians [1].

Despite the disparate in mental health hospitalisation rates of Aboriginal peoples, a suboptimal use of mental health services by Aboriginal peoples persists [4]. Barriers to care include poorly established partnership and communication among mental health services and Aboriginal peoples [5], poor understanding of the determinants of social and emotional well-being [6], cultural insensitivity [7], and persistent perceptions of mental health stigma [8]. As such, a goal of the Aboriginal mental health workforce is to engage their people and improve the social and emotional well-being of Aboriginal peoples [9]. Duties of the workforce may involve contact with consumers experiencing both emotional and spiritual illness along with distinct mental illness, which extends beyond the normal consumer-therapist relationship [10].

In fulfilling their roles, the Aboriginal mental health workforce can beneficially affect barriers to the use of mental health services in rural settings [11–13]. However, the effectiveness of such mental health specific roles in metropolitan settings is barely known, despite the fact that 75 % of Aboriginal peoples are located in non-rural areas [14]. Forty-eight percent of the Australian State of Victoria’s 37,991 Aboriginal population live in metropolitan Melbourne [15, 16]. Furthermore, urban settings attract diverse Aboriginal cultures from other geographical locations, which need to integrate with urban Aboriginal organisations and resources [17]. As such, there is a need for empirical evidence to support approaches outlined in the grey literature whereby services attempt to be physically available, affordable, appropriate, and culturally acceptable in meeting the needs of Aboriginal peoples [18].

In Victoria, the Aboriginal health reform priority ‘*fixing the gaps and improving the patient journey*’ has focused on improving Aboriginal people’s satisfaction with the care provided by hospitals and the transition between hospital and other health and social care agencies [19]. In 2013, the Northern Area Mental Health Service (NAMHS) initially obtained one-off funding for a Clinical Engagement Project which aimed to increase access and support for Aboriginal peoples and their families to the NAMHS. It was decided to pilot a 0.8 full time equivalent position of an Aboriginal Mental Health Liaison Officer (AMHLO). The AMHLO would be part of the acute mental health inpatient service and also work across the emergency department (ED) of the Northern Hospital, which is located at the same site and through which some mental health consumers are admitted. Given the unique development of such a role in an urban mental health service, this study was an attempt to describe how the AMHLO at the NAMHS may be able to improve Aboriginal people’s transition to mental health services. Specifically, the aims of this study were to; (1) describe the development of the AMHLO role in an urban setting and (2) describe stakeholder perceptions on how the role of the AMHLO impacts on the typical journey of Aboriginal consumers engaging with mental health services.

## Methods

### Study design

An illustrative case study was undertaken to meet the study aims with both quantitative and qualitative data. A case study investigates a complex intervention or approach within the real-life context in which it occurs [20]. An illustrative case study is primarily a descriptive study that typically uses one or two instances of an event to illustrate an intervention or approach, with a focus on the contextual elements of the case [21]. This method was chosen because of the paucity of knowledge about the AMHLO role in urban mental health settings. Ethical approval for the study was gained from the Melbourne Health Office for Research (QA/LNR 2013149).

### Data collection

Descriptive quantitative data were developed from informal written accounts of workload kept by the AMHLO over the first 9 months of the role. The database developed included the source of referral, a brief socio-demographic and clinical profile, and the service the Aboriginal consumer was referred to at discharge. However, the the primary emphasis in data collection was on qualitative data collected through semi-structured interviews with key stakeholders who had direct experience in interfacing with the role of the AMHLO. An independent approach was made to key stakeholders by the project officer overseeing the development of the role, to gain permission to be approached by the principal investigator (BM) to undertake informed consent to participate in the interviews. The principal investigator undertook a total of 11 interviews, each approximately one hour in length. Stakeholders approached included the AMHLO, managers, and clinicians who interfaced with, or were involved in developing the role.

The interview schedule included items which enquired about the conceptualisation, development, and implementation of the AMHLO role; perceptions of how the role assisted in the provision of mental health care for Aboriginal consumers and their families; views on changes in service provision since the establishment of the role; and perceived enablers and barriers to the role. An emphasis was placed on ‘narrative inquiry’ in all interviews [22]. Respondents were encouraged to relate stories that exemplified the items asked. This process allows description of the central phenomenon relative to the context of the respondent [23] and to facilitate a rich source of data for analysis. All interviews were conducted either in person or via telephone, digitally recorded, and transcribed. Interviews were undertaken across May and June 2014.

### Data analysis

Descriptive statistics were computed for Aboriginal peoples based on the database developed by the AMHLO. A thematic analysis of the qualitative data was undertaken using a general inductive approach on the descriptions of the development of the role and descriptions of the consumer journey through the service. A thematic analysis enables defensible analysis of qualitative data that may initially be varied raw text and allows it to be condensed into brief summaries [24]. Data were analysed through the processes of immersion, coding, creating categories, and identifying themes [25]. Interview data were transcribed verbatim, integrated with field notes, and independently read repeatedly by two researchers (BM & SF). Colour codes were developed though agreement among the researchers and categories were formed. Categories were collapsed to themes and examined for supporting quotes from the data [26, 27]. A total of 11 one-to-one interviews were held with a purposive sample of key stakeholders who were involved in either the development of the AMHLO role or who interfaced with and could describe the role. This included the voice of Aboriginal liaison roles (the AMHLO and a senior Aboriginal Health Liaison Officer from the general hospital); the voice of an Aboriginal manager role (a manager of an external Aboriginal health service provider); the voice of service managers (a mental health service manager, a mental health inpatient facility manager, and a mental health emergency service manager); and the voice of clinicians (a consultant psychiatrist, a psychiatric registrar, an inpatient nurse leader, an inpatient occupational therapist, and an inpatient social worker).

## Results

### Quantitative description - Profile of Aboriginal consumers interfacing with the AMHLO

The AMHLO received 37 referrals over a 9 month period (see Table 1). The major source of referral was from the ED (*n* = 18; 49 %). Seventy-three percent of referrals by the AMHLO on discharge were to community mental health teams (i.e., the youth early psychosis program, the Prevention and Recovery Care Service, and the Victorian Aboriginal Health Service).

**Table 1:** Sample Descriptors

		*n* (%)
Gender	Male	17 (46)
	Female	20 (54)
Legal Status	Voluntary	17 (46)
	Involuntary	20 (54)
Hx Admission	Yes	10 (27)
	No	27 (73)
Referral source	ED	18 (58)
	Acute inpatient mental health unit	11 (35)
	Self-referral	2 (7)
Discharge referral	Youth Early Psychosis Program	12 (32)
	Prevention and Recovery Care Service	8 (22)
	Victorian Aboriginal Health Service	7 (19)
	Other	10 (27)

NOTE: referral source missing data, *n* = 6

Other; the general public hospital, a secure mental health service, a private psychiatrist, a lawyer, and corrections officials

### Qualitative findings - Major themes

The analysis of the data on the development of the AMHLO role (aim 1) resulted in two themes themes; (1) realisation of the need to improve accessibility and (2) advocating for change. The themes highlighted the lack of engagement of Aboriginal peoples with the mental health service, the rationale for the AMHLO role, and the processes that allowed the conceptualisation and development of the role. The themes of the description of the AMHLO role (aim 2) were (1) the initiator: initiating access to the service, (2) the translator: brokering understanding among consumers and clinicians, (3) the networker: discharging to the community, and (4) the facilitator: providing cyclic continuity of care. The themes highlighted the multifaceted nature of the AMHLO role from service entry to discharge, the establishment of meaningful relationships, cultural sensitivity, and community engagement.

#### Development of the role

##### Realisation of the need to improve accessibility

The realisation of an increasing population of Aboriginal peoples in the northern suburbs of Melbourne was acknowledged as the catalyst for the need for an improved mental health service response. It was acknowledged that there was incongruence between the perceived mental health needs of the Aboriginal community and the numbers of Aboriginal consumers who were actually being seen by the area mental health service:

*“I saw some need, but we did not see large numbers of Aboriginal people coming to the unit. Sometimes they were not recorded properly… or people coming to the hospital were not using the services. So we did not think it was a large number. We did not question it.”* (Service managers voice)

Respondents commented on the inadequacy of mental health service delivery that was weighted towards clinical pragmatism rather than cultural need. They revealed that existing services were neither adequate nor sophisticated enough to meet the complex needs of Aboriginal consumers:

*“We did not have referral pathways. We kept on telling people (in the community) to rock up at times of emergency and good luck, or ring the crisis team. So that wasn’t really terrific.”* (Aboriginal manager voice)

##### Advocating for change

Alongside this realisation was a deep seated ethical commitment to address what was considered to be a social justice issue:

*“I think also individual workers including myself had a deep seated sense of wanting to do something. It’s not exactly shame, but it is part of that and we really genuinely wanted to do something good for Aboriginal people.”* (Service managers voice)

An existing relationship between the manager of an external Aboriginal health service and the senior Aboriginal Health Liaison Officer embedded in the general hospital attached to the mental health services cultivated the seeds for change. They had a long-standing vision of the potential benefits of an Aboriginal mental health worker position in assisting Aboriginal consumers. These individuals were committed to advocating for change:

*“So the number one key issue was how do you start advocating? So we sort of agreed that wherever we could advocate for the AMHLO, we would.* [The Aboriginal Health Liaison Officer] *did a fantastic job at that and I did whatever I could behind the scenes.”* (Service managers voice)

Strong relationships were also forged with the service manager of the mental health services. This was an enduring relationship based on mutual trust and respect:

*“It was about being open, flexible, and willing to listen… It was about listening and exchanging ideas, concerns, barriers, challenges… So the consultation is not tokenistic.”* (Aboriginal liaison voice)

These relationships coincided with opportunity through the Department of Health. The Department of Health were keen to initiate a number of Aboriginal Health Clinical Engagement Projects in Victoria to achieve systems change within hospitals to improve the health of Aboriginal peoples, through improved clinical engagement. Advocacy regarding the vision of an AMHLO in the mental health service coincided with a funding opportunity by the Department of Health. Funding was secured for a project to address systemic change to better address the needs of Aboriginal peoples. A project steering committee was established representing key stakeholders from NAMHS, the Victorian Aboriginal Health Service (VAHS), Aboriginal expertise in the general hospital mental health services, the Department of Health, and other local organisations. There was a strong commitment to undertake the work in partnership with Aboriginal colleagues and services. In this regard, the development of the AMHLO role was co-produced:

*“…so the wording of the position description, we did together. The wording of the ad, getting that out to the Aboriginal community, we did together. The interviews we did together. The referee checks - if we recruited* [the wrong person]*, then we are all in that together as well.”* (Service managers voice)

#### Description of the role

The AMHLO role was developed in an attempt to improve Aboriginal people’s transition to mental health services. The typical mental health clinical pathway from admission to discharge and referral can involve multiple assessments, treatment options, and discharge strategies. However, given the identified gap among mental health service availability and uptake, the typical clinical pathway for Aboriginal peoples, from service engagement to referral, only began to emerge with greater clarity after the AMHLO commenced at the service.

##### The initiator: Initiating access to the service

The AMHLO assisted in initiating the smooth entry of Aboriginal consumers to the mental health service through valued consultation with clinical staff. One point of entry for people being admitted to the mental health service was the ED. This required the AMHLO to have a presence in the ED and to establish relationships with mental health staff to ensure consultation occurred regarding Aboriginal peoples who presented in mental health emergencies. Significant gains were made in this regard:

*“The ED clinician rang me and said ‘I don’t want to admit her, come down have a chat with her and see what you think’. …The clinician said it was drug and alcohol, so I went and spoke with the client to see what was up. The minute that I had spoken with her she let me know that there was a lot going on in her life that impacted on her mental health. I went back to the clinician and said I recommend an admission. So she was admitted.”* (Aboriginal liaison voice)

Consultation also occurred with the AMHLO when Aboriginal peoples were being managed in the community, in order to prevent admission to hospital. This required a direct relationship with the Crisis Assessment and Treatment Team (CATT) and the ability to bring a cultural dimension and understanding which enlightened their management approach of Aboriginal peoples in mental health crisis:

*“We had a male patient who was a suicide risk. We called him to try and engage him to establish the level of risk and he perceived it to be interrogation… He did not want to engage. Normally it would mean we would have to escalate and insist on seeing the person. The AMHLO was actually able to contact him, was then able to talk to him about the CATT’s usual practice and procedures and explain what we were trying to do. The AMHLO was able to engage with him and bring him back into the loop.”* (Service managers voice)

##### The translator: Brokering understanding among consumers and clinicians

The primary focus of the AMHLO role is within an acute inpatient mental health unit. The role required developing trust with consumers to facilitate engagement and to provide support for Aboriginal consumers and their families. The cultural mismatch of world views and the dominant paradigm of the medical model within the complex and dynamic inpatient setting was seen as leading to potential oversight, misunderstanding, and miscommunication regarding cultural needs. The AMHLO was commonly described as the “interpreter” allowing consumers, their families, and clinicians to better align to address cultural need.

Although part of the clinical team, the AMHLO was not a case manager or key worker for Aboriginal consumers. The role was perceived as an “add on to”, rather than “instead of”, standard clinical practice. Interpretation to the clinical staff involved assisting clinicians to refine the planning of care to reflect a more holistic approach to service delivery, which incorporated a cultural dimension. The AMHLO involvement in clinical handover and clinical review enabled this interpretation and translation:

*“It just adds another layer of depth to what we are doing. I guess there is a whole lot of stuff around circumstances, language, culture, health and well-being, and we have some understandings of our work being culturally sensitive. But having* [the AMHLO] *there means that* [the AMHLO] *can answer anything that you need, to even the little things about what might be the best way to approach an issue.”* (Clinicians voice)

However, the role was not passive in merely enabling the ‘cultural literacy’ of clinicians. Interpretation readily flowed into advocacy on behalf of Aboriginal consumers:

*“This client had a lot of issues that she needed to sort out while she was in hospital. For example, she needed to get her car registered. This seems like a small thing but it was really important for this woman and the doctor did not want to give her leave. The AMHLO was able to give a different explanation as to why it was so important for this young woman to have a car. What her role in the family was. By giving that cultural explanation to the situation, we were able to advocate together to get the doctors to agree for her to have leave.”* (Clinicians voice)

This fine line between consultation on cultural need and advocacy extended to meeting the needs of the consumer’s family. The forum for such clarification was family meetings. Before the meetings, the AMHLO established and facilitated key family member involvement. During the meetings, the AMHLO supported the active involvement of the family in decision making.

##### The networker: Discharging to the community

Arranging discharge pathways and coordinating referrals and follow-ups for Aboriginal consumers was acknowledged as historically problematic by those interviewed. Organisational restrictions relating to the need for people to live in a particular catchment area resulted in inflexibility. This limitation had meant that Aboriginal people from other areas who may be visiting family were likely to miss out on services at discharge. However, the AMHLO’s cultural understanding provided a means of maintaining engagement post-discharge from the acute inpatient mental health unit:

*“Often when the* [Aboriginal] *patients come through they have a history somewhere else and that would be hard for us to access or know or understand what that is all about.”* (Clinicians voice)

The ability of the AMHLO to broker such engagement required an in-depth knowledge of referral opportunity in the community. Within mainstream community agencies, which were common discharge options initiated by clinical staff, advocacy by the AMHLO was required to prioritise the appropriateness of the placement for Aboriginal consumers. Some Aboriginal consumers were reluctant to engage with culturally specific services because of feelings of “shame” that people in the community would find out about their mental illness. The AMHLO was able to instill an awareness of the privacy and confidentiality associated with such engagement. The position has facilitated duel points of referral to both mainstream and culturally specific services, enabling the consumer to exercise choice by having post-discharge options:

*“I know* [the AMHLO] *has negotiated for an Aboriginal man an extended stay in a Prevention and Recovery Care service.* [The AMHLO] *advocated for that and he was also referred to a drug and alcohol program through email. The AMHLO is able to open up opportunity by being a good advocate.”* (Clinicians voice)

The mental health service had fickle historical relationships with Aboriginal specific services. The AMHLO developed relationships with such services through networking and created the opportunity for referral opportunity. This was facilitated through the bridge of a common cultural identity:

*“The AMHLO is great with liaison with the Victorian Aboriginal Health Service, which we have liaised with before but proved to be difficult.* [The AMHLO] *obviously has a greater understanding of the way they work ….* [The AMHLO] *is good at helping us get more access, liaising with them, linking with them, which we would not have perhaps done prior.”* (Clinicians voice)

##### The facilitator: Providing cyclic continuity of care

Acute inpatient mental health service engagement is generally time limited with clearly defined entry and exit points associated with admission, discharge, and referral. However, the AMHLO role transcended such time frames. Once the cultural link was established, engagement was maintained even when consumers were formally discharged from the service:

*“The cultural substance of this role is that a family or individual will make contact with you again. Almost all of them have made contact again after discharge but their file is closed. I asked my manger how I can record this for future account. I’m not going to say to a client who phones, ‘well you are not an in patient anymore, see you later’, because all the hard work I’ve done to build that rapport goes out the window. It’s a trust thing and once an Aboriginal person has a key Aboriginal worker they will always go to that worker if they need that service. They like to inform me of how they are travelling.”* (Aboriginal liaison voice)

## Discussion

Knowledge of the role of Aboriginal health worker role is primarily confined to studies in general health services [28]. Research on Aboriginal mental health workers predominantly focuses on the role in rural and remote areas [11–13]. The unique focus of the AMHLO role piloted at the NAMHS in the current illustrative care study allowed transition among an urban medical and mental health service, which is central to improving Aboriginal people’s satisfaction with the care provided [19]. The AMHLO was able to engage consumers with the mental health service and facilitate cultural understanding at times of such engagement, discharge, and integration with community services external to the NAMHS.

There is an emphasis in the literature on describing the role of Aboriginal health workers, with such positions being ‘cultural brokers’, who enable clearer communication of cultural specific needs to health professionals who then facilitate more holistic care [13, 28]. Elements of this brokerage were seen in the role of the AMHLO throughout the journey of Aboriginal consumers engaging with the mental health service and may be associated with grey literature highlighting the need for culturally acceptable health service [18]. The AMHLO focused on creating a greater understanding for the multidisciplinary team regarding culturally specific needs, at the point of entry and discharge, but especially during inpatient admission. However, meaningful interpersonal relationships were also established between the AMHLO, individual Aboriginal consumers, and their families. Such relationships were even maintained when Aboriginal consumers were formally discharged. Such strong interpersonal relationships are vital to the success of the Aboriginal health worker roles and are pivotal in fostering “vouching”, whereby the experience of positive engagement is disseminated throughout the Aboriginal community encouraging other members to engage [4]. This approach is instrumental to increase the modest volume of Aboriginal consumers interfacing with the role during its initial 9 months of operation. Furthermore, the AMHLO role in the current setting facilitated the creation of interpersonal relationships within a mental health service context that could be supported by the notion of an ‘appropriate’ health service [18].

Although such relationships do not translate to case management by the AMHLO, they allow the AMHLO to directly influence the planning of care within the multidisciplinary team. This is achieved by advocating for Aboriginal consumers and their families in clinical decision making forums in which Aboriginal peoples are either excluded or feel disempowered. Descriptions of the role of the AMHLO clearly demonstrated that this function was endorsed by the multidisciplinary team. This involvement in decision making goes beyond the emphasis on providing ‘cultural literacy’ to health professionals, normally associated with Aboriginal mental health worker roles [29, 30]. It enabled the AMHLO to be involved in assisting Aboriginal consumers to access services, in assisting in discharging such consumers to the community, and in providing continuity of care throughout the consumer’s pathway through the service and beyond.

Strong local managerial support is crucial for the development, implementation, and sustainability of Aboriginal mental health worker roles [9, 12, 29, 31]. The piloting of the AMHLO is dependent on on-going funding, which is not guaranteed. Given that the AMHLO role is in a pilot phase, flexibility is required to enable the role to self-define and expand. Such flexibility is also required to systematically address the multiple complex needs of Aboriginal consumers [12, 28, 32] and to allow the AMHLO role to become an integral part of the support and clinical treatment of Aboriginal consumers [33]. Nevertheless, the AMHLO role enabled flexibility in the clinical pathway for Aboriginal peoples and provided a resource for extended consultation and mental health literacy at the service, factors known to limit access to health services [18]. Furthermore, the AMHLO role may be used as an exemplar in an attempt to provide a culturally secure resource to engage Aboriginal peoples with mental health services. The method to implement such cultural services is barely known in health services [18], yet the processes of development and pragmatics of the AMHLO role in the current mental health setting may go some way to addressing a lack of empirical evidence.

### Limitations

This case study is limited to the study of the pilot role of an Aboriginal mental health worker in one metropolitan area mental health service with a relatively small but growing population of Aboriginal peoples. The description relies on a simple quantitative database and the purposive sampling of a small number of interviewees. As such, data may not represent the description of such roles in other mental health services throughout Australia. Furthermore, the voice of current Aboriginal consumers was not collected in the current study and as such their perceptions of the effectiveness of the AMHLO role remains unknown.

## Conclusions

The prioritising of ‘liaison’ in the title of the AMHLO is a misnomer. What became evident in this study is that liaison is only a part of the multiple tasks the urban AMHLO role fulfils. Multiple terms were used to capture this diversity including "advocate", "initiator", "interpreter", "cultural educator", "broker", "mediator", "facilitator", and "networker". These various descriptors demonstrate the considerable ability of the role to positively influence the lives of Aboriginal consumers and their families and improve engagement with health professionals in the mental health service in question. The vision of the AMHLO role is to improve the responsiveness and quality of service engagement provided by the mental health service to Aboriginal peoples and their families. This study does not attempt to evaluate this vision. However, the description of the intricacies of the role, paves the way for its future evaluation.

## Abbreviations

AMHLO: Aboriginal Mental Health Liaison Officer
CATT: Crisis Assessment and Treatment Team
ED: Emergency Department
NAMHS: Northern Area Mental Health Service
VAHS: Victorian Aboriginal Health Service
